# Adverse cardiac events of hypercholesterolemia are enhanced by sitagliptin in sprague dawley rats

**DOI:** 10.1186/s12986-024-00817-9

**Published:** 2024-07-30

**Authors:** Henry A. Palfrey, Avinash Kumar, Rashmi Pathak, Kirsten P. Stone, Thomas W. Gettys, Subramanyam N. Murthy

**Affiliations:** 1 Environmental Toxicology Department, Southern University and A&M College, Baton Rouge, LA 70813 USA; 2 Nutrient Sensing and Adipocyte Signaling, Pennington Biomedical Research Center, Baton Rouge, LA USA

**Keywords:** Cholesterol, Methionine, Cardiovascular, Sitagliptin

## Abstract

**Background:**

Cardiovascular disease (CVD) affects millions worldwide and is the leading cause of death among non-communicable diseases. Western diets typically comprise of meat and dairy products, both of which are rich in cholesterol (Cho) and methionine (Met), two well-known compounds with atherogenic capabilities. Despite their individual effects, literature on a dietary combination of the two in the context of CVD are limited. Therefore, studies on the combined effects of Cho and Met were carried out using male Sprague Dawley rats. An additional interest was to investigate the cardioprotective potential of sitagliptin, an anti-type 2 diabetic drug. We hypothesized that feeding a dietary combination of Cho and Met would result in adverse cardiac effects and would be attenuated upon administration of sitagliptin.

**Methods:**

Adult male Sprague-Dawley rats were fed either a control (Con), high Met (1.5%), high Cho (2.0%), or high Met (1.5%) + high Cho (2.0%) diet for 35 days. They were orally gavaged with an aqueous preparation of sitagliptin (100 mg/kg/d) or vehicle (water) from day 10 through 35. On day 36, rats were euthanized, and tissues were collected for analysis.

**Results:**

Histopathological evaluation revealed a reduction in myocardial striations and increased collagen deposition in hypercholesterolemia (HChol), responses that became exacerbated upon sitagliptin administration. Cardiac pro-inflammatory and pro-fibrotic responses were adversely impacted in similar fashion. The addition of Met to Cho (MC) attenuated all adverse structural and biochemical responses, with or without sitagliptin.

**Conclusions:**

Adverse cardiac outcomes in HChol were enhanced by the administration of sitagliptin, and such effects were alleviated by Met. Our findings could be significant for understanding or revisiting the risk-benefit evaluation of sitagliptin in type 2 diabetics, and especially those who are known to consume atherogenic diets.

## Introduction

Inflammation is considered as a cornerstone in many disease processes, particularly those of the cardiovascular system [1–4]. Although protective, unfavorable outcomes could occur if inflammation persists for a long period of time, as seen in the case of atherosclerosis [5]. Atherosclerosis is a type of arteriosclerosis (hardening of arterial walls) that is characterized by fibrofatty lesion formation in arterial walls. This causes arteries to become stenotic, impeding normal blood flow, and resulting in a multitude of downstream adverse effects [5]. From the onset of the atherosclerotic process to advanced stages, where complete plaque formation is present in arterial walls, which is a hallmark of cardiovascular disease (CVD), the expression of pro-inflammatory (e.g., tumor necrosis factor alpha, TNFα; interlukin-1 beta, IL-1β; etc.) and other biochemical indicators are commonly observed [6]. Essentially, it is biochemical processes like inflammation or oxidative stress that precede adverse structural changes, as seen in fibrosis [7].

Western diets are believed to contribute to CVD as they largely consist of compounds like sugars, cholesterol (Cho), sodium, and saturated fats among others [8–11]. Additionally, Cho and methionine (Met) are atherogenic and are found in large quantities in meat, poultry, and dairy products [12, 13]. Where approximately 70% of Cho is synthesized *de novo*, the dietary Cho contributes to about 30% of total body Cho [14–16]. It is, however, the overconsumption of Cho in the diet that has long been debated as a causative factor for CVD [17–19]. Initial reports of Cho as a contributing factor in CVD stem from results of the Framingham heart study of the 1960s [20]. Furthermore, elevated dietary Cho has also been associated with pro-inflammatory signaling in adipocytes, a situation that can adversely affect the heart in obese and diabetic patients [21, 22].

Methionine is an essential amino acid that serves as a methyl group donor in DNA, protein, and other methylations [23, 24]. Defects in Met metabolism, deficiencies of vitamins B6, B12 or folate, or increased consumption could result in the elevation of an intermediate compound, homocysteine (Hcy), in circulation and result in hyperhomocysteinemia; a noted risk factor for CVD [25]. Kilmer McCully, a pioneer of the Hcy theory, demonstrated that Hcy damages tissues by stimulating the release of cytokines, cyclins, and other mediators of inflammation and cell division [26]. Troen and coworkers have also reported an atherogenic effect of methionine [27]. Conversely, dietary Met restriction has been demonstrated to produce beneficial effects like increasing the longevity, improving insulin sensitivity and lipid profile, and enhancing metabolic flexibility [28–30].

Strong evidence has surfaced in recent years that highlights an association between non-alcoholic fatty liver disease (NAFLD) and increased CVD risk [31]. In fact, atherosclerotic CVD has been considered as the main cause for mortality in patients diagnosed with NAFLD [31]. The underlying mechanisms for the relationship between NAFLD and CVD are believed to be incompletely understood; however, inflammation, endothelial dysfunction, and dyslipidemia are documented as significant risk factors [32]. Our lab previously observed hepatic inflammatory and oxidative stress responses in hypercholesterolemia (HChol), an observation that was exacerbated by sitagliptin administration [33, 34]. Sitagliptin (Januvia) is a type 2 anti-diabetic drug currently in clinical use for the management of hyperglycemia, via dipeptidyl peptidase-4 (DPP-4) inhibition [35]. Independent of its hypoglycemic effect, sitagliptin has been shown to provide multiple health benefits such as attenuating heart and kidney failure and helping to improve neurological function [36–38]. Based on our previous data, we advanced our studies to investigate the cardiac effects of feeding a dietary combination of Cho and Met and evaluate the cardioprotective potential of sitagliptin. Therefore, *we hypothesized that feeding a high Cho + high Met diet would have an additive effect on the cardiac inflammatory and oxidative stress responses, and administration of sitagliptin would alleviate such effects.*

## Materials and methods

### Animals and diets

All animal experiments were performed according to the National Institutes of Health Guide for Care and Use of Experimental Animals. The protocol was approved by the Institutional Animal Care and Use Committee of the LSU Pennington Biomedical Research Center (PBRC) in Baton Rouge, LA. Adult male Sprague-Dawley rats weighing 250–270 g were obtained from Envigo RMS, Inc. (Indianapolis, IN). Purina #5001 Chow containing 25.05% carbohydrate, 24.1% protein and 11.4% fat and supplemented with 0.5% cholic acid and 2.0% maltose dextrin was used for the control (Con) diets. Dyets, Inc. (Bethlehem, PA) prepared the experimental diets by enrichment of the Con diet with 1.5 Met %, 2.0% Cho, and 1.5 Met % + 2.0% Cho (MC). Rats were housed individually in cages with standard bedding in a temperature and humidity-controlled room with a 12-hr day/night cycle for acclimatization for one week. Food and water were provided *ad libitum.*

### Experiments

In the 1st experiment, rats were weight-matched and divided into four dietary groups (Con, Met, Cho, MC; *n* = 7 per group) and fed for 35 days. After a 4-hour fast on day 36, they were euthanized by CO_2_ inhalation (for respiratory arrest) followed by the collection of blood and heart tissue for analysis. In the 2nd experiment, rats were weight-matched and assigned to Con (*n* = 14), Met (*n* = 7), Cho (*n* = 7) and MC (*n* = 7) groups. On day 10, half the Con and all rats in Met, Cho, and MC were orally gavaged with an aqueous suspension of *sitagliptin (100 mg/kg/day)*. The remaining Con rats were orally gavaged with vehicle (water) to validate a null effect of drug with a normal diet. The diet and drug regimen continued until the end of the experiment (day 35). After a 4-hour fast on day 36, they were euthanized by CO_2_ inhalation (for respiratory arrest) followed by the collection of blood and heart tissue for analysis. In a 3rd experiment, rats were weight-matched then assigned to Con, Cho, and MC groups (*n* = 16 per group); the single Met group was omitted. This experiment was conducted to authenticate findings of the 1st and 2nd experiments where diet and drug effects were assessed independently. On day 10, half the rats in each group were orally gavaged with vehicle, while the remaining half were administered sitagliptin (100 mg/kg/day) by oral gavage. The diet and drug regimen continued for 25 days. After a 4-hour fast on day 36, animals in each grouping were euthanized by CO_2_ inhalation (for respiratory arrest) followed by the collection of blood and heart tissues for biochemical analysis and histopathological evaluation.

### Sample collection

Basal (fasting) blood samples were collected prior to the start of each experiment via retro-orbital puncture under 2.0% isoflurane anesthesia, while terminal (fasting) blood samples were collected by cardiac puncture following CO_2_ euthanasia. Serum was separated from whole blood and stored at -80 °C for subsequent analysis. A segment of the heart tissue encompassing the left and right ventricles was processed for histopathological evaluation by the Cell Biology and Bioimaging Core of PBRC (https://www.pbrc.edu/research-and-faculty/core-services/cell-biology-and-bioimaging-core-section/). The remaining heart tissue was snap frozen in liquid nitrogen and stored at -80 °C for additional analysis.

### Measurement of body composition

Body weight and body composition were measured weekly for all rats in each experiment. Time domain-nuclear magnetic resonance (NMR) spectroscopy (Bruker Minispec, Billerica, MA) was used to measure body composition (lean mass & adiposity). Calibration of the NMR instrument was achieved using appropriate fat, lean mass, and water standards per the manufacturer’s protocol.

### Metabolomics

Serum from all rodents in each experiment underwent metabolomic analysis at the Biological and Small Molecule Mass Spectrometry Core facility at the University of Tennessee, under the direction of Dr. Shawn Campagna (https://chem.utk.edu/facilities/biological-and-small-molecule-mass-spectrometry-core-bsmmsc/).

### Histopathology

Masson’s trichrome staining was performed to assess collagen deposition in the heart tissue from each experiment. Heart samples were fixed in 10% neutral buffer formalin and processed on a TissueTek VIP 6 Vacuum Infiltration Processor. Following fixation, they were embedded in paraffin and 5 μm sections were obtained for histopathological evaluation. For trichrome staining, heart slides were first deparaffinized then rehydrated through a series of descending alcohol washes (100%, 95%, 70%). Slides were then washed (in distilled water) and re-fixed by incubation in Bouin’s solution (75mL Picric acid-saturated; 25 mL formaldehyde-37%; 5mL glacial acetic acid) for 1 h at 56 °C. The purpose of re-fixation was to improve staining quality. A working solution prepared from two Weigert’s Iron Hematoxylin stock solutions was now used to stain the slides, after which, slides were rinsed (placement under tap water for 10 min), washed (in distilled water), and stained once more by placement in a Biebrich scarlet acid fuchsin solution for 10 min. To highlight collagen fibers, slides were placed in a phosphomolybdic-phosphotungstic acid solution for 10–15 min or until collagen did not appear red in color. Without rinsing, the slides were transferred to an aniline blue solution and stained for 5 min. Subsequently, the slides were rinsed (distilled water) and rapidly dehydrated through 95% ethyl alcohol and absolute ethyl alcohol then cleared in xylene and mounted. For viewing and evaluation, the slides were scanned using a Hamamatsu Nanozoomer Digital Pathology system (Hamamatsu City, Japan).

### Immunohistochemistry

To authenticate adverse biochemical responses related to structural integrity and function, an H&E counterstain for cardiac troponin-I (cTn-I) was performed on heart sections from the 3rd experiment. To begin, slides were deparaffinized and placed in a pressure cooker and incubated for 20 min at 100 °C. Subsequently, they were allowed to cool, then rinsed (deionized water). Endogenous peroxidase activities were inactivated in 3% H_2_O_2_ in TBS for 12 min at 4 °C. Non-specific antibody binding sites were blocked, and slides were incubated with the primary antibody Troponin I (C-4): sc-133,117 (Santa Cruz) overnight at 4 °C; 1:500 dilution. After incubation, the slides were washed three times in 1X TBST at 3 min per wash. Secondary detection was performed by incubating the slides for 1 h at room temperature using Goat-anti-mouse IgG2a antibody HRP. Next, the slides were washed then incubated with 3,3’-Diaminobenzidine for 5–10 min and washed once more in deionized water. Lastly, the slides were treated with hematoxylin, dehydrated, and mounted with a coverslip. For viewing and evaluation, the slides were scanned using a Hamamatsu Nanozoomer Digital Pathology system (Hamamatsu City, Japan). Prior to staining, positive & negative controls were established to ensure antibody-specific binding.

### RNA isolation and quantitative real-time PCR

Total RNA from heart tissue was isolated using RNeasy mini kit (Qiagen, Germantown, MD, USA) according to the manufacturer’s protocol. Quantification of RNA was performed using a NanoDrop spectrophotometer (ThermoFisher Scientific, Waltham, MA, USA). Two micrograms of total RNA were reverse transcribed using oligo-(dT) 20 primers and M-MLV reverse transcriptase from Promega (Madison, WI) to synthesize complementary DNA. Gene expression was measured by real-time polymerase chain reaction (StepOne Real‐Time PCR System; Applied Biosystems, Foster City, CA, USA) by measurement of SYBR Green.Primer sequences are provided in Table 1. All mRNA samples were run in duplicate and fold change in the target gene expression compared to the expression of control genes by comparative threshold cycle (Ct) method. The mRNA expression data of target genes were normalized to the expression of a house-keeping gene cyclophilin A, expression levels were calculated using 2 – ΔΔCt method and expressed as fold change of normal control group.

### Measurement of cardiac proteins

Enzyme-linked immunosorbent assay (ELISA) kits were used to measure TNF-a and IL-1β (R&D Systems, cTN-I (Abcam; Cambridge, MA), and transforming growth factor beta1 / TGFβ1 (Elabscience; Houston, TX).

### Statistical analyses

One-way analysis of variance (ANOVA) for multiple comparisons was performed with diet or sitagliptin (1st and 2nd experiment) as the main effect followed by post-hoc analysis using Tukey correction for multiple comparisons, while two-way ANOVA was utilized for analyzing the main effects of diet and sitagliptin treatment (3rd experiment). Data is presented as the mean ± SE. *P* values of 0.05 or less were regarded as statically significant. Analysis was conducted using GraphPad Prism version 8.0.2 (San Diego, California).

**Table 1:** Sequence of primers used for RT-qPCR

Target Gene	Sequence
*CypA*	
Forward Reverse	TATCTGCACTGCCAAGACTGAGTG CTTCTTGCTGGTCTTGCCATTCC
*cTn-I*	
Forward Reverse	CACCTCAAGCAGGTGAAGAA TCTTTCGGCCTTCCATTCC
*rFabp*	
Forward Reverse	CGGTACCTGGAAGCTAGTGG TCATCTGCTGTGACCTCGTC
*Il1β*	
Forward Reverse	CAAGCAACGACAAAATCCCTG GACAAACCGCTTTTCCATCTTC
*Lox1*	
Forward Reverse	CCCACAAGTCACAGACTCTTC CACACACTCACACACACAAATAC
*αSma*	
Forward Reverse	GCTCCTCCAGAACGCAAATA CAGCTTCGTCATACTCCTGTTT
*Tgfβ1*	
Forward Reverse	AGAGCCCTGGATACCAACTA CAACCCAGGTCCTTCCTAAAG
*Tnfα*	
Forward Reverse	AGACCCTCACACTCAGATCA GTCTTTGAGATCCATGCCATTG

## Results

### Effect of atherogenic diets on changes to cardiac biochemical parameters in male SD rats

**Fig. 1:**
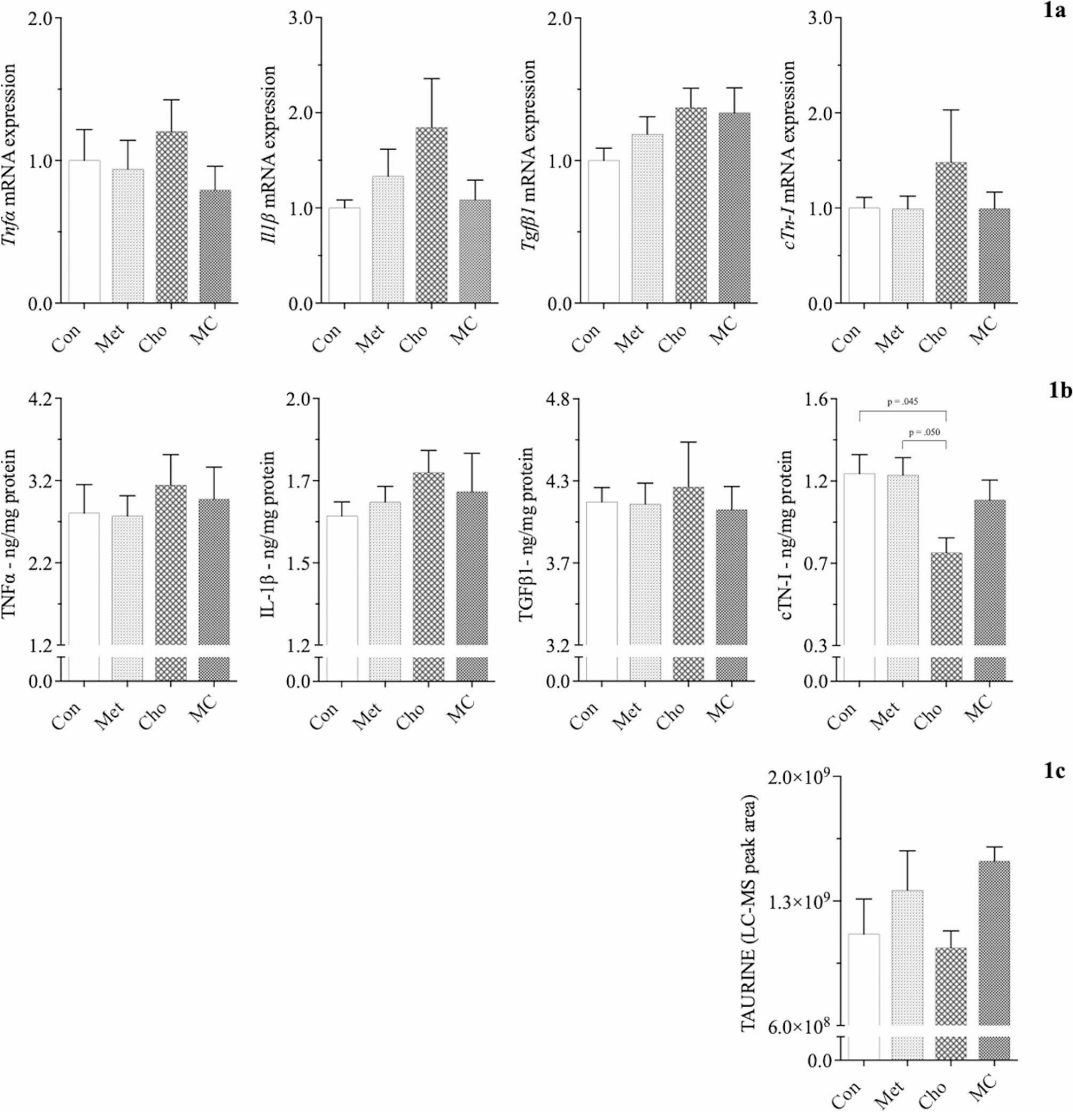
Effect of atherogenic diets on the expression of cardiac biomarkers in male Sprague-Dawley rats. Rodents were fed either a Con, high Met, high Cho, or high Met + high Cho (MC) diet *ad libitum* for 35 days. Gene expression of pro-inflammatory (*Tnfα, Il1β*) and pro-fibrotic (*Tgfβ1*) markers are shown (**1a**), along with their protein levels (**1b**) and serum taurine (**1c**). Values are presented as mean ± SE; *n* = 7

In comparison to Met and MC diets, Cho-feeding resulted in a reduction in cTN-I protein (∼ 34%) as compared to Con (normal) feeding; Fig. 1b. This response was the sole parameter showing a statistically significant change in the 1st experiment. This warranted further investigation for showing a diet-induced effect in the heart given the importance of the protein in regulating cardiac function, and since it was hypothesized that such a response would be cause by the combination diet. Although non-significant, MC-feeding was shown to attenuate the reduction of cTN-I protein, increase serum taurine (biomarker of antioxidation), and reduce non-significant increases in pro-inflammatory and pro-fibrotic mRNA and protein expression; Figs. 1a-1c. Interestingly, *cTn-I* mRNA expression was increased by Cho-feeding, however, the response was not statistically significant. As it relates to our hepatic studies by Kumar et al. (2020), the adverse effects of Cho-feeding (only) were far more impactful to the liver, resulting in statistically significant increases to hepatic oxidative stress genes. The observed responses in the current and former (hepatic) experiment were independent of body weight, body composition, and blood glucose levels, which we previously reported as being similar among diet groups [33, 34].

### Effect of sitagliptin on changes to cardiac biochemical parameters in male SD rats

In the 2nd experiment, the cardioprotective potential of sitagliptin was investigated. Rats fed Con, Met, Cho, and MC diets were administered sitagliptin (100 mg/kg/day) by oral gavage; Con-S, Met-S, Cho-S, MC-S. An additional Con group was added to validate the null effect of drug with a normal diet - here, rats were gavaged with vehicle (water); Con-V. Ultimately, relative comparison was to Con-V for determining adverse effects as the difference in values between Con-V and Con-S were minimal for any of the parameters measured.

**Fig. 2:**
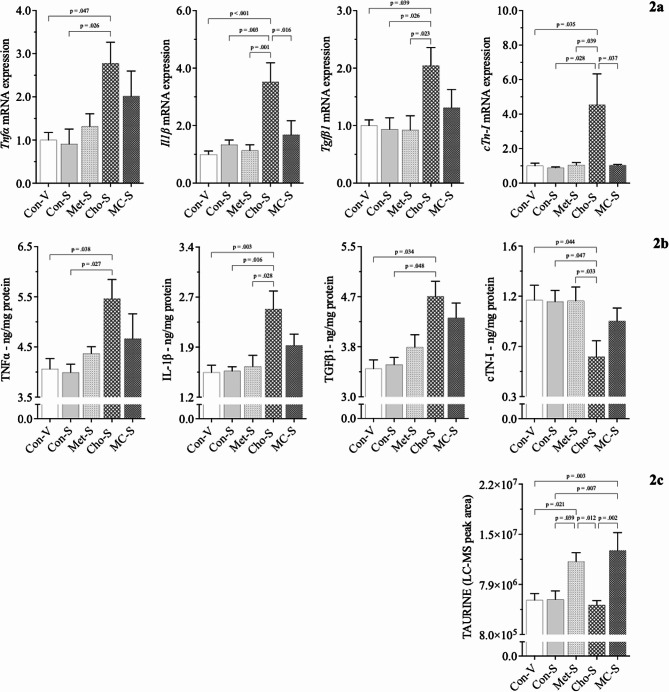
Effect of sitagliptin on the expression of cardiac biomarkers in male Sprague-Dawley rats fed atherogenic diets. Rats were fed either a Con, high Met, high Cho, or high Met + high Cho (MC) diet ad libitum for 35 days. Day 10 through 35, half the Con and all rats in Met, Cho, and MC groups were administered an aqueous suspension of sitagliptin (100 mg/kg/day) by oral gavage; Con-S, Met-S, Cho-S, MC-S. The remaining Con-fed rats were gavaged with vehicle (water); Con-V. Relative mRNA expression of *Tnfα*, *Il1β*, *Tgfβ1*, and *cTn-I* are shown (**2a**), along with TNFα, IL-1β, TGFβ1, and cTn-I protein levels (**2b**) and serum taurine (**2c**). Values are presented as mean ± SE; *n* = 7

In the 1st experiment, cTN-I protein was significantly decreased in heart tissue as a result of Cho feeding. A response that was exacerbated (by approximately 10%) with sitagliptin being administered to Cho-fed rats; Fig. 2b. On the contrary, *cTn-I* mRNA expression was significantly elevated 4-fold with sitagliptin administration in Cho-fed rats (Fig. 2a) as compared to < 1-fold increase without the drug. Sitagliptin administration resulted in increases of *Tnfα, Il1β*, and *Tgfβ1* mRNA expression in Cho-fed rats being statistically significant, along with TNFα, IL-1β, and TGFβ1 protein levels being significantly elevated as well; Figs. 2a, 2b. Rats fed the combination diet (MC) that were administered sitagliptin experienced (i) a reduction in *Il1β* and *cTn-I* mRNA expression and (ii) increased serume taurine, both of which are statistically significant; Figs. 2a, 2c. mRNA expression for *Tnfα* and *Tgfβ1*, along with TNFα, IL-1β, TGFβ1, and cTn-I protein, were attenuated as well in MC (+ sitagliptin) but were non-significant; Figs. 2^2^, 2^2^. Met-feeding with and without sitagliptin administration was shown to essentially have a null effect on nearly all parameters measured thus far, having mRNA expression and protein levels similar to that of Con-fed rats. Serum taurine is the sole parameter that was significantly elevated as a result of Met-feeding and sitagliptin administration; Fig. 2c. Similar to our hepatic studies by Kumar et al. (2020), sitagliptin administration has shown to exacerbate those adverse responses observed in Cho-feeding. The effects of sitagliptin herein are independent of body weight, body composition, and blood glucose levels, which we previously observed as being similar among groups [33, 34].

### Effects of atherogenic diets and sitagliptin on cardiac structural and biochemical changes in male SD rats

Following independent investigations on the effect of diet and sitagliptin in male SD rats, a 3rd experiment was conducted to further examine and corroborate those previous findings. Since Met feeding (alone) was shown to essentially have a null effect on increasing adverse reponses in the heart with and without sitagliptin, it was omitted from this experiment.

**Fig. 3:**
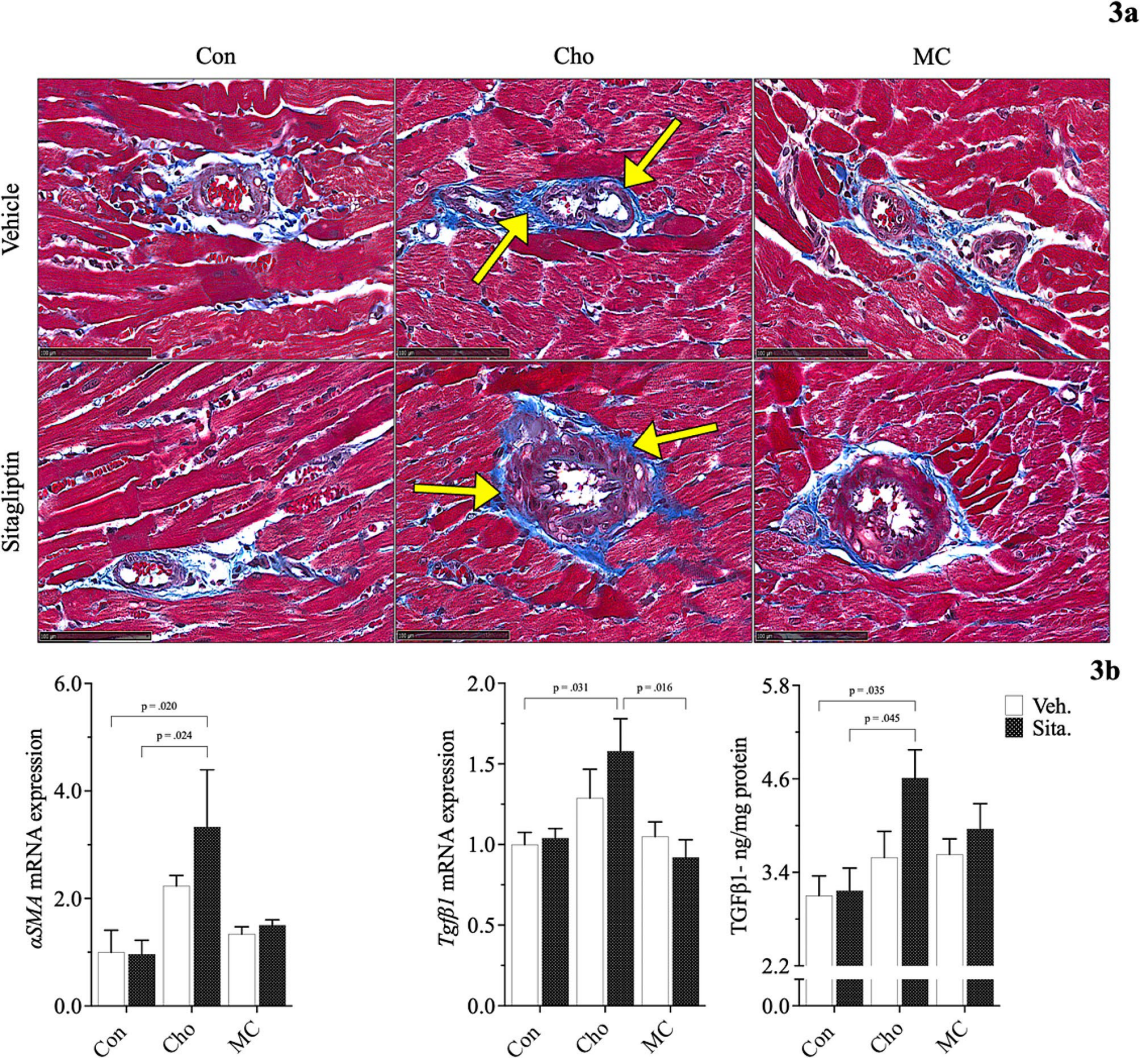
Effects of diet and sitagliptin on structural and biochemical parameters of fibrosis. Adult male Sprague-Dawley rats were fed either a Con, high Cho, or MC diet ad libitum for 35 days. Day 10 through 35, half the rats in each group were administered an aqueous suspension of sitagliptin (100 mg/kg/day) by oral gavage, while the remaining half were gavaged with vehicle (water). Representative photomicrographs of Masson’s trichrome staining (**a**; 40X) of heart tissue showing collagen deposition (arrows), along with associated biomarkers of fibrosis (**b**), are depicted. Images show increased collagen deposition as a result of Cho feeding with and without sitagliptin administration. Values are presented as mean ± SE; *n* = 8

**Fig. 4:**
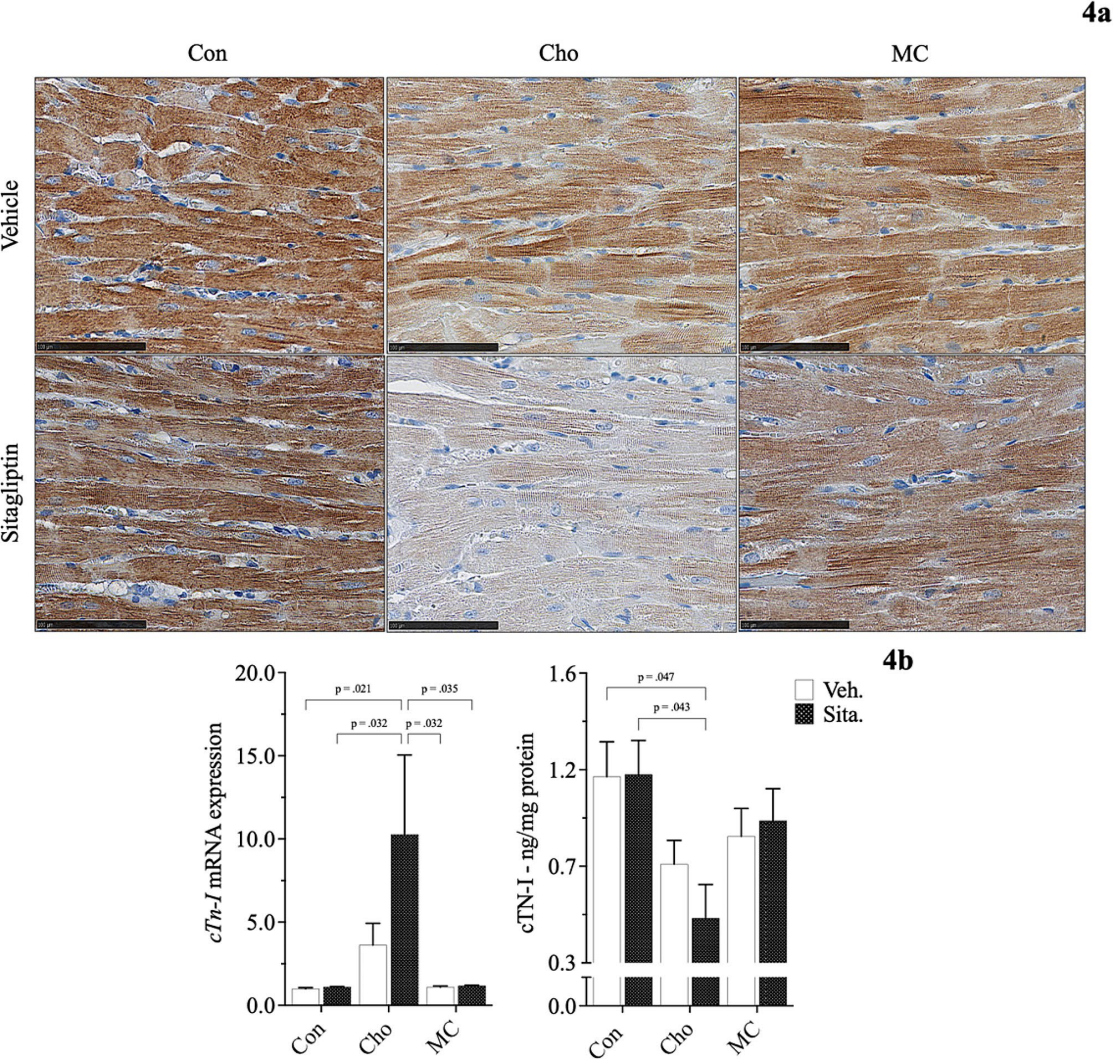
Effects of diet and sitagliptin on cardiac troponin-I expression in male SD rats. Rats were fed either a Con, high Cho, or MC diet ad libitum for 35 days. Day 10 through 35, half the rats in each group were administered an aqueous suspension of sitagliptin (100 mg/kg/day) by oral gavage, while the remaining half were gavaged with vehicle (water). Representative photomicrographs of H&E counterstain for cTn-I (**a**; 40X) showing a reduction in myocardial striations (i.e., loss of cTN-I protein) is shown, along with *cTn-I* mRNA expression and cTN-I protein levels (**b**). Values are presented as mean ± SE; *n* = 8

Histopathological evaluation revealed both an increase in collagen deposition surrounding blood vessels in the heart and a reduction in myocardial striations (i.e., loss of cTN-I protein) in rats fed Cho. Administration of sitagliptin appears to exacerbate the effects of both to some degree; Figs. 3a, 4a. Since alpha smooth muscle cctin (αSMA) is commonly used as a marker of myofibroblast formation, and myofibroblasts function to upregulate collagens, it helps to serve as a marker of fibrosis alongside the TGFβ1 and others [39]. mRNA expression of *αSma* and *Tgfβ1*, along with TGFβ1 protein, were significantly elevated in Cho-fed rats who were administered sitagliptin; Fig. 3b. Alterations to myocardial striations were confirmed by measuring cTN-I protein in heart tissue, which was significantly reduced following sitaglitpin administration to Cho-fed rats; Fig. 4b.

**Fig. 5:**
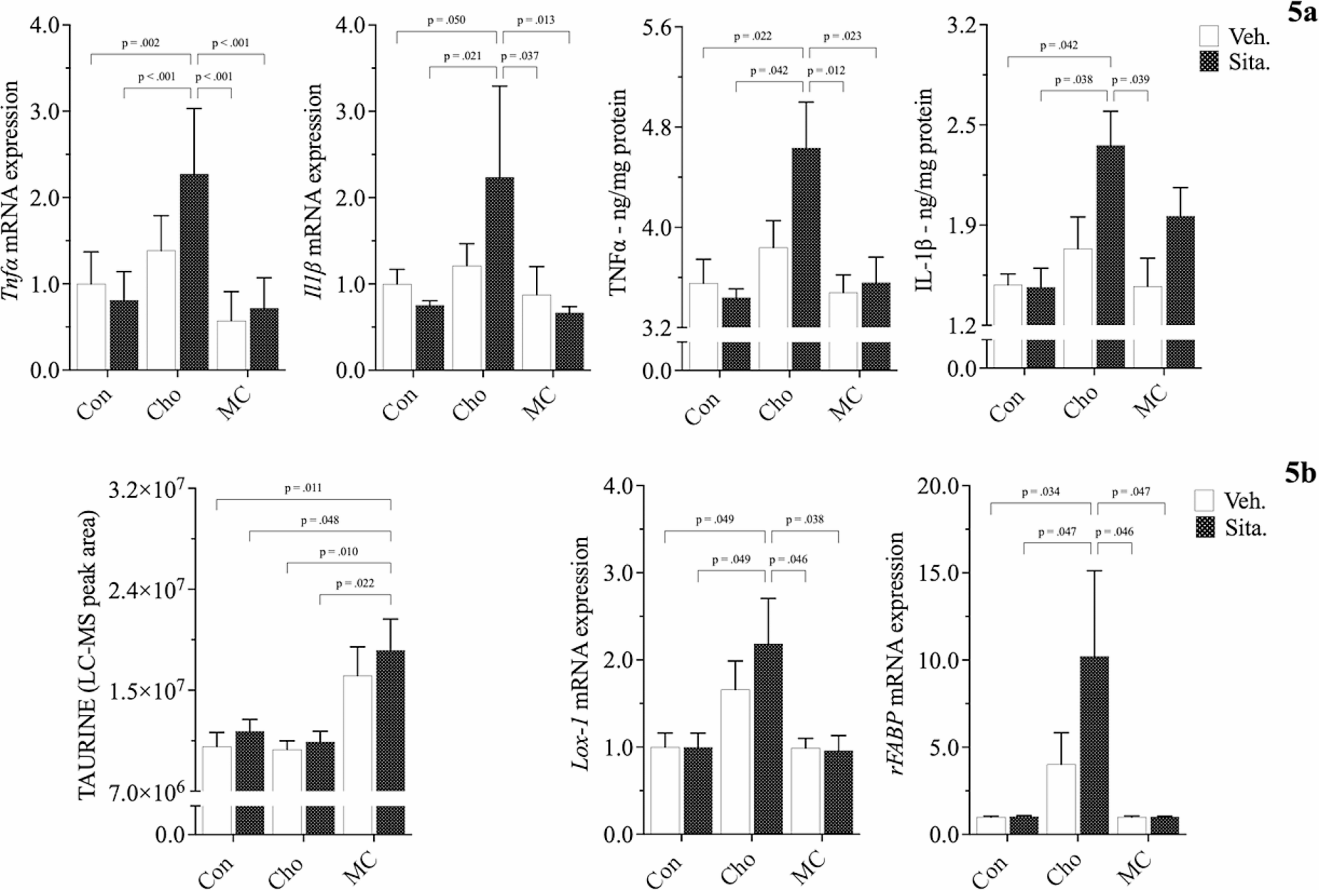
Effects of diet and sitagliptin on cardiac biomarkers of inflammation, Cho transport, and antioxidation. Male Sprague-Dawley rats were fed either a Con, high Cho, or MC diet ad libitum for 35 days. Day 10 through 35, half the rats in each group were administered an aqueous suspension of sitagliptin (100 mg/kg/day) by oral gavage, while the remaining half were gavaged with vehicle. Relative mRNA expression and protein levels of pro-inflammatory indicators in HChol (+/- sitagliptin) are shown (**a**), along with biomarkers of Cho/fatty acid transport and antioxidation (**b**). Values are presented as mean ± SE; *n* = 8

Pro-inflammatory markers (TNFα, IL-1β) and those related to Cho & fatty acid transport (Lectin-like oxidized low-density lipoprotein - LOX1; Rat fatty-acid-binding protein - rFABP) were increased as well in HChol, and exacerbated with sitagliptin administration; Figs. 5a, 5b. Lectin-like oxidized low-density lipoprotein receptor is of importance because it is a scavenger receptor involved in oxidized low-density lipoprotein (oxLDL) uptake from the blood, after which oxLDL ultimately contributes to arterial plaque formation [40]. Rat fatty-acid-binding protein is part of a family of transport proteins that distribute fatty acids and other lipophilic compounds across intra- and extracellular membranes [41].

All adverse responses observed in HChol with and without sitagliptin administration were attenuated in MC-fed rats with and without sitagliptin. Serum taurine was the sole biomarker increased in MC feeding, both with and without the drug; Fig. 5b. All responses were independent of body weight, body composition, and blood glucose levels, which we previously reported as being similar among groups [33, 34].

## Discussion

It is well known that CVD and NAFLD are major public health concerns globally with high morbidity and mortality. Both have been associated with elevated circulating levels of Cho and Hcy, an intermediate in Met metabolism. Though endogenous as well as dietary Cho sources contribute to circulating levels of Cho, non-pharmaceutical management (i.e., dietary approaches) is well-known for lowering Cho levels. With the emerging controversy about the role of Cho in CVD, it remains evident that elevated blood Cho can greatly affect liver function, as the liver is a main processing center for Cho [42]. Also, there are reports that point to the liver being affected by HChol more than the heart and that chronic liver disease can have a direct impact on heart function [43].

The approach in our animal studies were to feed a dietary excess of Cho and Met independently, but moreso in combination since studies on the combined effects are not as numerous. We also aimed to investigate the cardioprotective potential of sitagliptin, which is documented as improving cardiac function and ejection fraction. Sitagliptin is used in the pharmacotherapy of glucose management in type II diabetics and has displayed positive effects (e.g., weight lowering, reduction of inflammation / oxidative stress / fibrotic responses) independent of glucose-lowering [44–46].

Adverse biochemical events occurring in the heart can result in conditions like arrhythmias, myocardial infarction, and heart failure eventually [47–49]. Diagnosis of such events requires a concerted effort that usually commences with cardiac function tests. This involves imaging techniques (e.g., echocardiograms, magnetic resonance imaging scans, computed tomography scans, nuclear cardiac stress test, coronary angiogram or left heart catheterization, X-rays, etc.), biopsies, and/or serological assays [50–54]. As it relates to a clinical diagnosis of acute myocardial infraction, elevated blood cTN-I levels are an indicator for such. Elevated cTN-I in circulation serves as an indicator of cardiac injury; thus, we considered its assessment as a reasonable starting point for determining adverse cardiac outcomes in our heart-related study [54, 55]. We assessed cardiac function primarily by histopathological evaluation and quantification of cTN-I protein in heart tissue. Initially, we observed a significant decrease of cTN-I protein in heart tissue as a result of Cho-feeding. An effect that became exacerbated upon sitagliptin administration. This observed effect of diet and sitagliptin was a *novel* finding contrary to our central hypothesis, which was to observe high Met + high Cho feeding having an additive effect on inducing adverse cardiac outcomes and for seeing an alleviation by sitagliptin administration. Instead, Cho-feeding with sitagliptin administration were shown as the culprits leading to unfavorable responses in the heart. Han et al. (2018) saw a reduction of cTN-I protein in heart tissue as well, as a result of feeding male SD rats a high-fat, high Cho diet for 14 and 28 days [56]. Interestingly, we observed an elevated 4-fold change in *cTn-I* mRNA expression in HChol (+ sitagliptin), an effect that is possibly due to a compensatory response, as demonstrated by Sasse et al. (1993) [57]. Studies by Packer (2018) also show a positive correlation between DPP-4 inhibitor use and adverse cardiac events, citing their ability to cause and/or worsen heart failure [58,59]. Rouse et al. (2014) and Shahbaz et al. (2018) correlate sitagliptin use with pancreatic injury and acute hepatitis, respectively [60,61].

Cho feeding in our study was shown to increase collagen deposition surrounding blood vessels in the heart, as well as within the interstitial spaces. Sitagliptin appeared to exacerbate that effect to some degree. Notably, cardiac fibrosis is classified as either endomyocardial fibrosis, infiltrative & reactive interstitial fibrosis, or replacement fibrosis [62]. HChol (+/- sitagliptin) seems to have resulted in a form cardiac perivascular fibrosis, which is characterized by collagen accumulation around blood vessels [63, 64]. This is known to precede or coincide with *reactive interstitial fibrosis* - collagen accumulation that causes expansion of cardiac interstitial spaces with minor cardiomyocyte loss [63, 64]. Although the increase in collagen deposition by Cho may not seem unique, as this was demonstrated by Han et al. (2018), the seemingly sitagliptin exacerbation is interesting. A reason for such an observation could be due to sitagliptin’s interaction with Cho to affect some factor in TGFβ signaling. Three isotypes of TGFβ have been identified in mammals (TGFβ1, TGFβ2, TGFβ3) and many animals studies identify type 1 as the “master regulator” that promotes fibrotic development in several tissues [65–67]. TGFβ1 utilizes several signaling pathways to elicit a variety of actions (e.g., autophagy, differentiation, apoptosis, and cellular proliferation). However, it is the Smad-dependent (canonical) pathway that is most noted as resulting in fibrosis [65–67]. Sitagliptin likely stimulates the canonical pathway in some way, but this remains to be proven. Like cardiac smooth muscle, myofibroblasts in heart tissue express αSMA and are abundantly located in the thick myocardial layer. Myofibroblasts help to regulate various functions such as matrix deposition & degradation and growth-factor secretion [68]. mRNA expression of *αSma* and *Tgfβ1*, along with TGFβ1 protein, was increased in HChol as well and exacerbated with sitagliptin administration.

Insight into the underlying molecular mechanisms by which the adverse structural responses were seen in HChol, with and without sitagliptin administration, was investigated in our study. Biochemical changes are those precede structural changes in all cell types and are part of processes like oxidative stress and inflammation [69, 70]. Such changes could, in fact, be sex-specific as well, as Marques et al. (2018) discovered an association between increased IL-6 and C-reactive protein expression and the development of interstitial myocardial fibrosis in men [71]. Additional literature also points to TNFα and IL-1/6 being key mediators for myocardial alterations [55]. We observed significantly increased *Tnfα* and *Il1β* mRNA expression in the heart tissue of rats fed Cho and administered sitagliptin; TNFα and IL-1β protein were significantly increased as well. Both biomarkers are well-known to stimulate pro-inflammatory signaling [72–74]. Previously, we measured serum Cho in the hepatic studies performed by Pathak et al. (2019), showing an approximate 100% increase in rats fed Cho (+/- sitagliptin) as compared to Con-fed rats (+/- sitagliptin). With increased availability of Cho in the blood, this increases the formation / transport of low-density lipoprotein, which can become oxidized and transported into tissues, via LOX1 [39]. This process is disadvantageous because it can result in plaque formation in arterial walls, and complete organ dysfunction ultimately [1–6]. Both *Lox-1* and *rFabp* mRNA were greatly increased in our rats fed Cho and administered sitagliptin. In our previous hepatic studies by Pathak et al. (2019) and Kumar et al. (2020), the magnitude of adverse hepatic responses was greater than what is being demonstrated in the current study. An explanation could simply be related to the liver’s increased exposure to compounds in the blood since it primarily functions to metabolize, transport, and filter compounds that are absorbed and placed into circulation [75]. In either case of the liver or heart, sitagliptin was shown to enhance the adverse biochemical and structural responses seen in HChol.

The addition of Met to the Cho diet did not produce an additive effect as originally hypothesized. In fact, it proved beneficial by way of attenuating all adverse cardiac responses in HChol, bringing them closer to normal levels. This was interesting because Met restriction is outlined in literature as being beneficial, however, our results were on the contrary. We did not notice any obvious disruptions to Met metabolism, as Hcy levels and gene expression of the Met-metabolizing enzymes were unaffected. Unexpectedly, Met- and MC-feeding led to increased serum taurine levels, even moreso with the administration of sitagliptin. Taurine is a compound with anti-oxidative and anti-inflammatory effects, both of which could have contributed to the beneficial responses we observed [76]. In addition to taurine, there are other intermediates in Met metabolism that are documented to elicit multiple health benefits, i.e., anti-oxidation & -inflammation, vasodilation [77, 78]. Additional studies are needed to better understand this.

In summary, our study provides insight into the effects of DPP4-inhibitor use and atherogenic diets on the biochemical and structural changes in the heart. We demonstrated that sitagliptin administration exacerbates adverse cardiac responses seen in HChol, while also revealing the beneficial potential of high dietary Met to attenuate such effects. To gain more understanding of this diet-drug relationship, additional studies are needed. The beneficial aspect of high dietary Met observed in our study merit mechanistic understanding for exploring future therapeutic options considering the public health relevance of CVD and are thus translational.

## Data Availability

No datasets were generated or analysed during the current study.

## Abbreviations

ANOVA: Analysis of variance
Cho: Cholesterol
Con: Control
cTN-I: Cardiac troponin-I
CVD: Cardiovascular disease
DPP-4: Dipeptidyl peptidase-4
ELISA: Enzyme linked immunosorbent assay
rFABP: Rat Fatty-acid-binding Protein
HChol: Hypercholesterolemia
Hcy: Homocysteine
HRP: Horseradish peroxidase
IgG2a: Immunoglobulin 2 alpha
IL-1β: Interlukin-1 beta
IL-6: Interlukin-6
LDL: Low-density lipoprotein
LOX1: Lectin-like oxidized low-density lipoprotein receptor-1
MC: Methionine + Cholesterol
Met: Methionine
mRNA: Messenger ribonucleic acid
NAFLD: Non-alcoholic fatty liver disease
NMR: Nuclear magnetic resonance
oxLDL: Oxidized low-density lipoprotein
PCR: Polymerase chain reaction
SE: Standard error of the mean
αSMA: Alpha-smooth muscle actin
SYBR: Synergy Brands
TBS: Tris buffered saline
TBST: Tris buffered saline – Tween 20
TGFβ1: Transforming growth factor beta 1
TNFα: Tumor necrosis factor alpha
RNA: Ribonucleic acid
